# Substantial Inter-Subject Variability in Blood Pressure Responses to Glucose in a Healthy, Non-obese Population

**DOI:** 10.3389/fphys.2017.00507

**Published:** 2017-07-18

**Authors:** Cathriona R. Monnard, Benoît Fellay, Isabelle Scerri, Erik K. Grasser

**Affiliations:** ^1^Department of Medicine/Physiology, University of Fribourg Fribourg, Switzerland; ^2^Laboratoire HFR, Central Laboratory, Hôpital Fribourgeois–Cantonal Hospital Fribourg Fribourg, Switzerland

**Keywords:** beat-to-beat, body composition, sugars, insulin sensitivity, OGTT

## Abstract

**Aim:** A large inter-subject variability in the blood pressure (BP) response to glucose drinks has been reported. However, the underlying factors remain elusive and we hypothesized that accompanying changes in glucose metabolism affect these BP responses.

**Methods:** Cardiovascular and glycemic changes in response to a standard 75 g oral-glucose-tolerance-test were investigated in 30 healthy, non-obese males. Continuous cardiovascular monitoring, including beat-to-beat BP, electrocardiographically deduced heart rate and impedance cardiography, was performed during a 30 min baseline and continued up to 120 min after glucose ingestion. Blood samples were taken at baseline, 30, 60, 90, and 120 min for the assessment of glucose, insulin and c-peptide. Additionally, we evaluated body composition by using validated bioelectrical impedance techniques.

**Results:** Individual overall changes (i.e., averages over 120 min) for systolic BP ranged from −4.9 to +4.7 mmHg, where increases and decreases were equally distributed (50%). Peak changes (i.e., peak averages over 10 min intervals) for systolic BP ranged from −1.3 to +9.5 mmHg, where 93% of subjects increased systolic BP above baseline values (similar for diastolic BP) whilst 63% of subjects increased peak systolic BP by more than 4 mmHg. Changes in peak systolic BP were negatively associated with the calculated Matsuda-index of insulin sensitivity (*r* = −0.39, *p* = 0.04) but with no other evaluated parameter including body composition. Moreover, besides a trend toward an association between overall changes in systolic BP and total fat mass percentage (*r* = +0.32, *p* = 0.09), no association was found between other body composition parameters and overall BP changes.

**Conclusion:** Substantial inter-subject variability in BP changes was observed in a healthy, non-obese subpopulation in response to an oral glucose load. In 63% of subjects, peak systolic BP increased by more than a clinically relevant 4 mmHg. Peak systolic BP changes, but not overall BP changes, correlated with insulin sensitivity, with little influence of body composition.

## Introduction

Cardiovascular disease (CVD) is one of the leading causes of morbidity and mortality in European and many other countries worldwide (Santulli, 2013). In this context, a leading risk factor for CVD mortality is high blood pressure (BP), which accounts for > 40% of CVD-related deaths worldwide (Danaei et al., 2014). In individuals with type 2 diabetes mellitus (T2DM), elevated BP further increases the risk of CVD (Turner et al., 1993), whilst T2DM itself imposes a risk of coronary heart disease mortality equivalent to that of a prior myocardial infarction (Juutilainen et al., 2005). Moreover, a recent study confirmed a time dependent and additive effect of BP and increasing blood glucose levels on the development of T2DM complications (Stratton et al., 2006). It is noteworthy that even a state of impaired glucose tolerance, a condition that usually precedes T2DM, substantially elevates the risk for CVD (DeFronzo and Abdul-Ghani, 2011). Therefore, the possibility arises that even early stage perturbations in glucose metabolism may affect the cardiovascular system.

It is generally agreed that in response to glucose ingestion the resulting increase in blood glucose levels induces a rapid increase in plasma insulin, which dose-dependently increases cardiac output (by increasing stroke volume and heart rate) and attenuates systemic vascular resistance (Baron and Brechtel, 1993). In this context, the decrease in vascular resistance is the driving force for increases in stroke volume and heart rate in order to counterbalance a potential drop in BP. This assertion is supported by previous findings from our group, which show increased heart rate, stroke volume, cardiac output, and double product (as a marker of cardiac oxygen demand Van Vliet and Montani, 1999) in response to glucose ingestion, whilst total peripheral resistance was found to decrease (Brown et al., 2008a; Grasser et al., 2014); these effects being accompanied by either no change (Brown et al., 2008a), or a small increase in BP (Grasser et al., 2014).

Recognized as a standard diagnostic test for glucose intolerance and diabetes, the oral-glucose-tolerance-test (OGTT) is a simple method that provides pertinent information on individual glucose metabolism. Despite the abundance of information available on glucose and insulin responses to an OGTT, little is known about concomitant changes in BP. In young and healthy adults, ingestion of a glucose containing drink, in opposition to fructose, has repeatedly been shown to have little impact on postprandial systolic- and diastolic BP (Brown et al., 2008a; Grasser et al., 2014) although a retrospective analysis revealed a large inter-subject variability in the response to glucose ranging from −2.3 to +8.3 mmHg (averaged response over 60 min post-drink after subtracting baseline values) (Grasser et al., 2014), an observation, which, to our best knowledge, has not yet been prospectively investigated. Moreover, the factors underlying the effect of this potential inter-subject variability in BP in response to glucose remain elusive. One potential explanation could be that this inter-subject variability is the result of altered insulin action, which may perturb BP regulation.

We hypothesized firstly (i) that postprandial BP regulation in response to a standardized glucose load exhibits substantial inter-subject variability and, secondly, (ii) that these BP responses are affected by accompanied glycemic responses. Therefore, we evaluated, in 30 healthy male, non-obese adults, beat-to-beat cardiovascular and glycemic blood parameter responses to a standardized 75 g oral glucose drink. Additionally, and given the important influence of body composition on the cardiovascular and metabolic systems, we evaluated anthropometric and adiposity parameters in order to explore their potential association with overall and peak changes in BP.

## Materials and methods

### Subjects

Study participants were recruited from the university population and surrounding institutions through advertisements. All subjects completed a medical questionnaire, a dietary intake questionnaire (including caffeine, caffeinated soft drinks and alcohol habits), and had a physical exam to ensure their suitability for inclusion in the study. Subjects recruited to the study were familiarized with all cardiovascular monitoring equipment and experimental procedures to avoid possible period effects. Subjects were considered eligible for inclusion if they were healthy, non-smokers, Caucasian men, 165–200 cm in height, 20–45 years old with a body mass index (BMI) ranging from 18.5 to 29.9 kg/m^2^. Exclusion criteria included any medical condition that could interfere with the measured variables, e.g., cardiovascular-, gastrointestinal-, neurological-, and overt metabolic disorders. Moreover, subjects taking medication (either for acute or chronic illnesses), competition athletes, smokers, overtly sedentary, those with a fear of needles or who had in the past adverse reactions to cannulation were excluded. Finally, participants diagnosed with T2DM based on the following criteria in response to the OGTT were excluded: showing either (a) fasting glucose levels ≥7.0 mmol/L (126 mg/dL) (American Diabetes Association, 2009), or (b) 2-h glucose ≥ 11.1 mmol/L (200 mg/dL) (American Diabetes Association, 2009). Written informed consent to participate in the study was obtained from all subjects prior to their first test. The study protocol complied with the Declaration of Helsinki and received ethical approval from the Commission cantonale d'éthique de la recherche sur l'être humain (Canton de Vaud, Switzerland).

### Study design

All experiments took place in a quiet, temperature-controlled (22 ± 1°C) laboratory and started at 08.00 a.m. following a 12-h overnight fast. Subjects were requested to avoid alcohol, caffeine and physical activity for 24 h prior to the test, and to use public transportation to arrive to the laboratory on the morning of the experiment. On arrival to the laboratory, subjects were asked to empty their bladders if necessary and to sit in a comfortable armchair. Electrocardiography (ECG)/Impedance electrodes were positioned together with upper arm and BP cuffs placed on the fingers. Electrode strips were placed at the neck and thoracic regions; the latter specifically at the mid-clavicular at the xiphoid process level (Grasser et al., 2009) and an 18-gauge 1.3 × 33-mm one-way *Teflon* catheter was inserted in the left cubital vein (not possible in one subject). Following a variable period for attainment of cardiovascular and metabolic stability (approximately 30 min), a baseline recording was then made for 30 min. Following this, a baseline blood sample (10 mL) was drawn, which was followed by ingestion of the glucose drink [75 g of anhydrous D-glucose, dissolved in 300 mL water at room temperature (22°C)] over 4 min. Cardiovascular recordings then continued for another 120 min and blood samples (10 mL, respectively) were drawn every 30 min thereafter, for up to 120 min post-drink. In order to avoid boredom during the study, subjects were allowed to watch documentaries on a TV screen placed in front of them.

### Cardiovascular measurements

A Task Force Monitor (CNSystems, Medizintechnik, Graz, Austria) was used to measure hemodynamic changes over time (systolic BP, diastolic BP, RR-interval (RRI), and stroke volume) (Girona et al., 2014). Data were sampled at a rate of 1,000 Hz and stored on a hard disc for later analysis. Continuous BP monitoring followed the Penaz principle from either the index or middle finger of the right hand and was calibrated to oscillometric brachial BP measurements on the contralateral arm without perturbations caused by the calibration signal (Gratze et al., 1998). We used height-adjustable tables for reliable horizontally aligned placement of BP cuffs, i.e., table heights were adjusted to the height of the right atrium (forth-intercostal space), in order to avoid misleading BP readings based on deviations from heart level. Moreover, an adaptive cushion was placed on top of each table where the subject's forearms rested comfortably throughout the study. BP cuff sizes were chosen according to the upper arm circumference. Cardiac stroke volume, Heather index (a marker for positive inotropy of the heart), and thoracic fluid volume were derived through impedance cardiography measurements (Hill and Merrifield, 1976; Sherwood et al., 1998). Impedance cardiography, in which changes in thoracic impedance are converted to reflect changes in thoracic fluid content/volume over time, were performed based on the original Kubicek (Kubicek et al., 1970) approach, but using an improved estimate of thoracic volume (Fortin et al., 2006).

### Blood sample measurements

Each blood collection comprised an initial 1 mL blood draw, which was immediately discarded due to clotting, and a subsequent 10 mL blood sample, which was used for analysis. Samples were collected in appropriate K2E (EDTA) BD vacutainers (Becton, Dickinson Allschwil, Switzerland) and were processed and centrifuged according to the manufacturer's instructions to separate plasma from whole blood. Aliquots were stored in 2 ml cryovials and plasma samples for glucose analysis were immediately frozen and stored at −80°C; plasma samples for the analysis of insulin and C-peptide were stored at −20°C. After thawing the samples, assays were performed according to the manufacturer's instructions (SystemRoche/Hitachi cobas, Roche Diagnostics, Basel, Switzerland): (i) Glucose by the reference method with hexokinase Glucose HK Gen.3 (cobas c 501, Roche Diagnostics); (ii) C-peptide by ECLIA technology (cobas e 601, Roche Diagnostics). Insulin was measured by using an ELISA assay kit (Mercodia, Uppsala, Sweden) in accordance with the manufacturer's instructions. Phlebotomy and blood sample processing were carried out in accordance with institutional safety requirements for the handling of human biological specimens.

### Anthropometric and body composition measurements

Anthropometric measurements included (i) standing height using a mechanical column scale with integrated stadiometer (Seca model 709, Hamburg, Germany) and (ii) body weight using an electronic scale (Tanita Corporation, Amsterdam, The Netherlands). Body mass index was calculated as the ratio of weight (kg) and height squared (m^2^). Waist circumference (WC) was measured in a standing position using a non-stretch tape. Body composition was measured using two devices: (i) multi-frequency bioelectrical impedance analyzer (BIA; Inbody 720, Biospace Co., Ltd, Seoul, Korea) for the assessment of total fat mass (percentage and kg), fat free mass (kg), skeletal muscle mass (kg) and (ii) a dual-frequency BIA device (ViScan AB-140, Tanita) for the assessment of trunk (abdominal) fat percentage (Hunma et al., 2016). The ViScan technique has been validated against magnetic resonance imaging for the prediction of abdominal fat percentage (Browning et al., 2010).

### Data collection/processing

Beat-to-beat values of RRI, heart rate, systolic BP and diastolic BP, stroke volume, cardiac output, total peripheral resistance, Heather index, double product, and thoracic fluid content were averaged every 10 min during baseline and every 10 min during the 120 min post-drink period. Overall changes were calculated as averages over the entire 120 min measurement period with baseline values subtracted. To derive peak changes, changes over the 120 min measurement period were divided into 12 × 10 min averages and the maximum response in any one of these 10 min average intervals was taken as the peak change. Heart rate was calculated from the appropriate RRI. Cardiac output was computed as the product of stroke volume and heart rate. Total peripheral resistance was calculated as [mean BP/cardiac output], where mean BP was calculated from diastolic BP and systolic BP as follows: [mean BP = diastolic BP + 1/3 (systolic BP-diastolic BP)]. Double product was calculated as [heart rate × systolic BP]. Homeostatic model assessment for insulin resistance (HOMA-IR) was calculated as [fasting glucose (mmol/L) × fasting insulin (mIU/L)]/22.5 (Matthews et al., 1985) and the Matsuda-Index of insulin sensitivity (ISI-Mat) as [10,000/square root of (fasting glucose × fasting insulin) × (mean glucose × mean insulin during OGTT)] (Matsuda and DeFronzo, 1999).

### Statistical analysis

**Figure 1:**
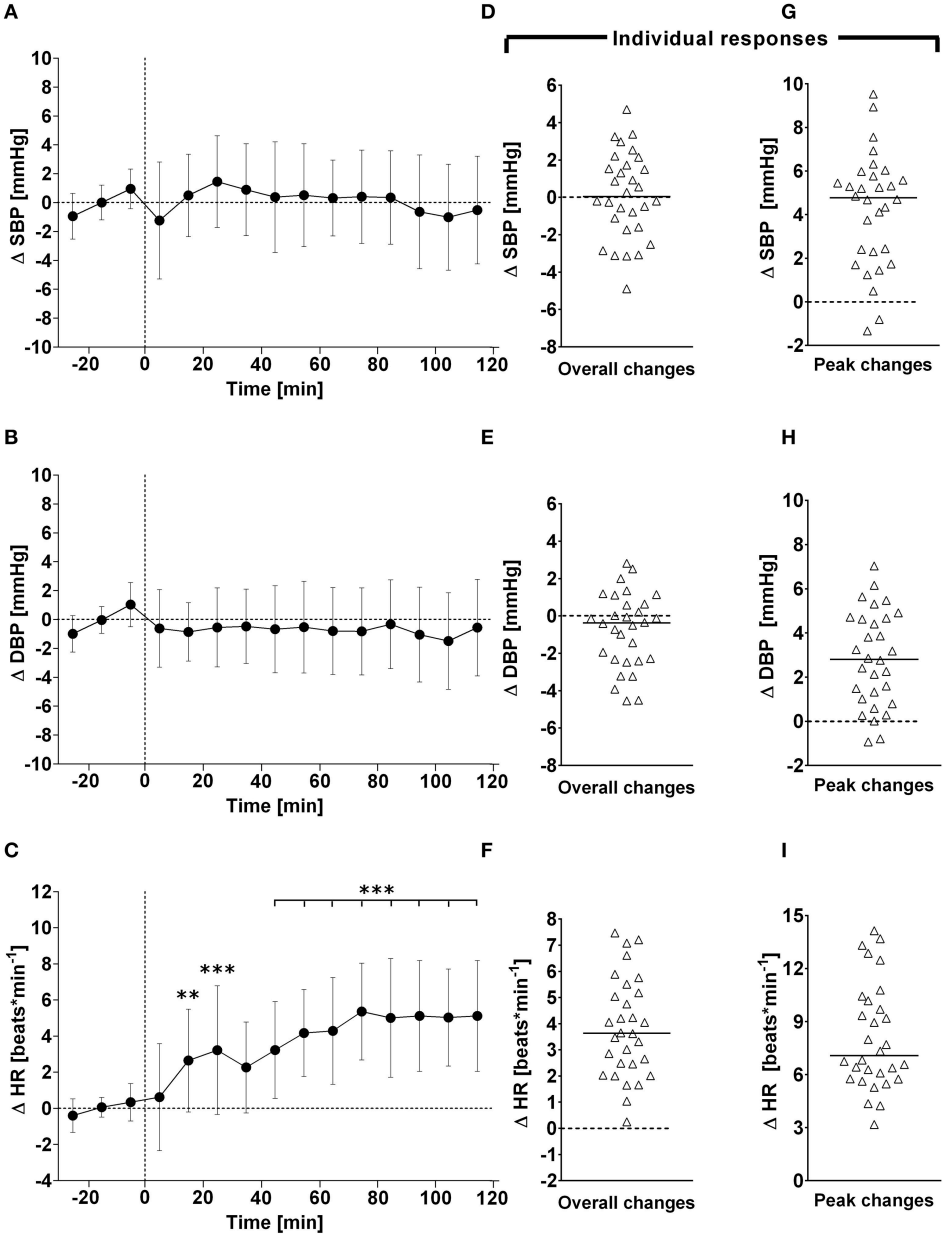
**(A–C)**: Time course of changes (Δ) from baseline in systolic blood pressure (SBP), diastolic blood pressure (DBP), and heart rate (HR), respectively. Closed circles (•) represent averaged beat-to-beat data over 10 min intervals, which were subtracted from each baseline level and presented as deltas. Data recorded during the 4 min glucose ingestion period were excluded from the analysis, and Time 0 denotes the resumption of continuous cardiovascular measurements after subjects have finished the oral glucose drink. **(D–F)** represent overall (i.e., averages over 120 min with baseline values subtracted) changes in SBP, DBP, and HR relative to baseline. **(G–I)** represent peak (i.e., derived from the maximum response averaged over a 10 min interval) changes in SBP, DBP, and HR relative to baseline. ^**^P < 0.01 and ^***^P < 0.005 represent statistically significant differences over time from baseline values (left). values are reported as means ± SD, whilst right data are presented as a scatter dot plot with a median (bold horizontal dashed line).

**Figure 2:**
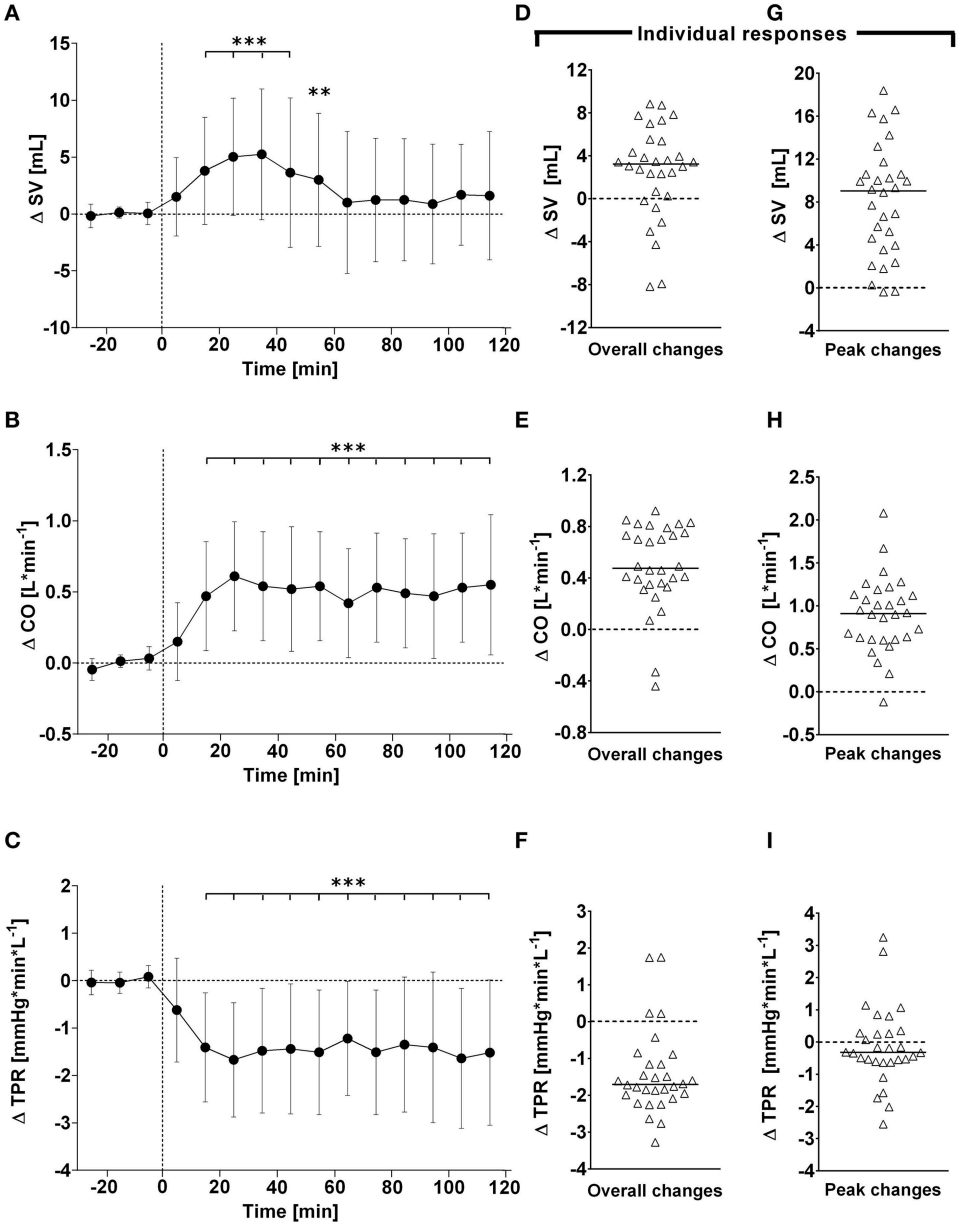
**(A–C)**: Time course of changes (Δ) from baseline in stroke volume (SV), cardiac output (CO) and total peripheral resistance (TPR), respectively. Closed circles (•) represent averaged beat-to-beat data over 10 min intervals, which were subtracted from each baseline level and presented as deltas. Data recorded during the 4 min glucose ingestion period were excluded from the analysis, and Time 0 denotes the resumption of continuous cardiovascular measurements after subjects have finished the oral glucose drink. **(D–F)** represent overall (i.e., averages over 120 min with baseline values subtracted) changes in SV, CO, and TPR relative to baseline. **(G–I)** represent peak (i.e., derived from the maximum response averaged over a 10 min interval) changes in SV, CO, and TPR relative to baseline. ^**^P < 0.01 and ^***^P < 0.005 represent statistically significant differences over time from baseline values (left). Left values are reported as means ± SD, whilst right data are presented as a scatter dot plot with a median (bold horizontal dashed line).

**Figure 3:**
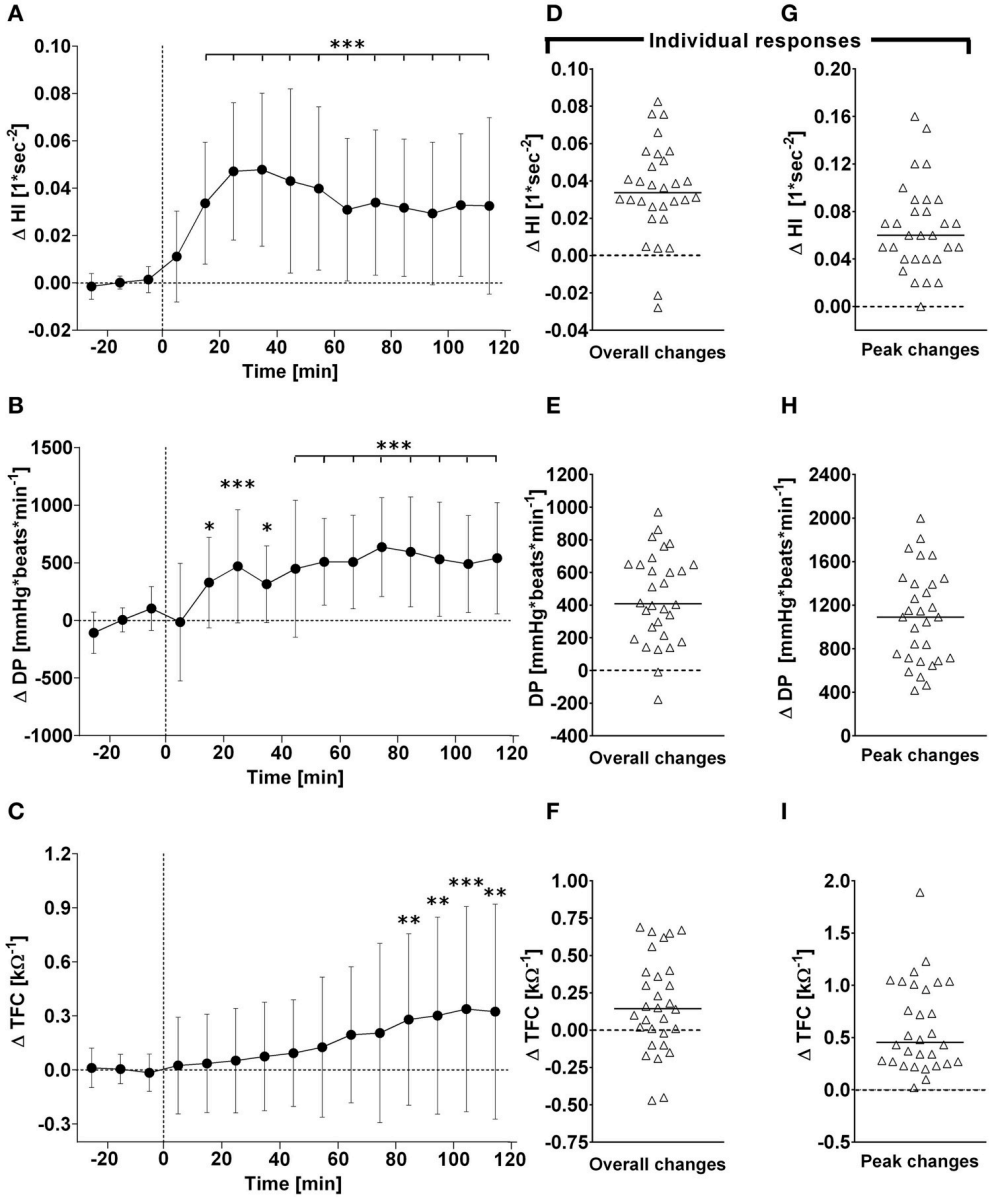
**(A–C)**: Time course of changes (Δ) from baseline in Heather index (HI), rate pressure double product (DP), and thoracic fluid content (TFC), respectively. Closed circles (•) represent averaged beat-to-beat data over 10 min intervals, which were subtracted from each baseline level and presented as deltas. Data recorded during the 4 min glucose ingestion period were excluded from the analysis, and Time 0 denotes the resumption of continuous cardiovascular measurements after subjects have finished the oral glucose drink. **(C–E)** represent overall (i.e., averages over 120 min with baseline values subtracted) changes in HI, DP, and TFC relative to baseline. **(E–G)** represent peak (i.e., derived from the maximum response averaged over a 10 min interval) changes in HI, DP, and TFC relative to baseline. ^*^P < 0.05, ^**^P < 0.01 and ^***^P < 0.005 represent statistically significant differences over time from baseline values (left). Left values are reported as means ± SD, whilst right data are presented as a scatter dot plot with a median (bold horizontal dashed line).

**Figure 4:**
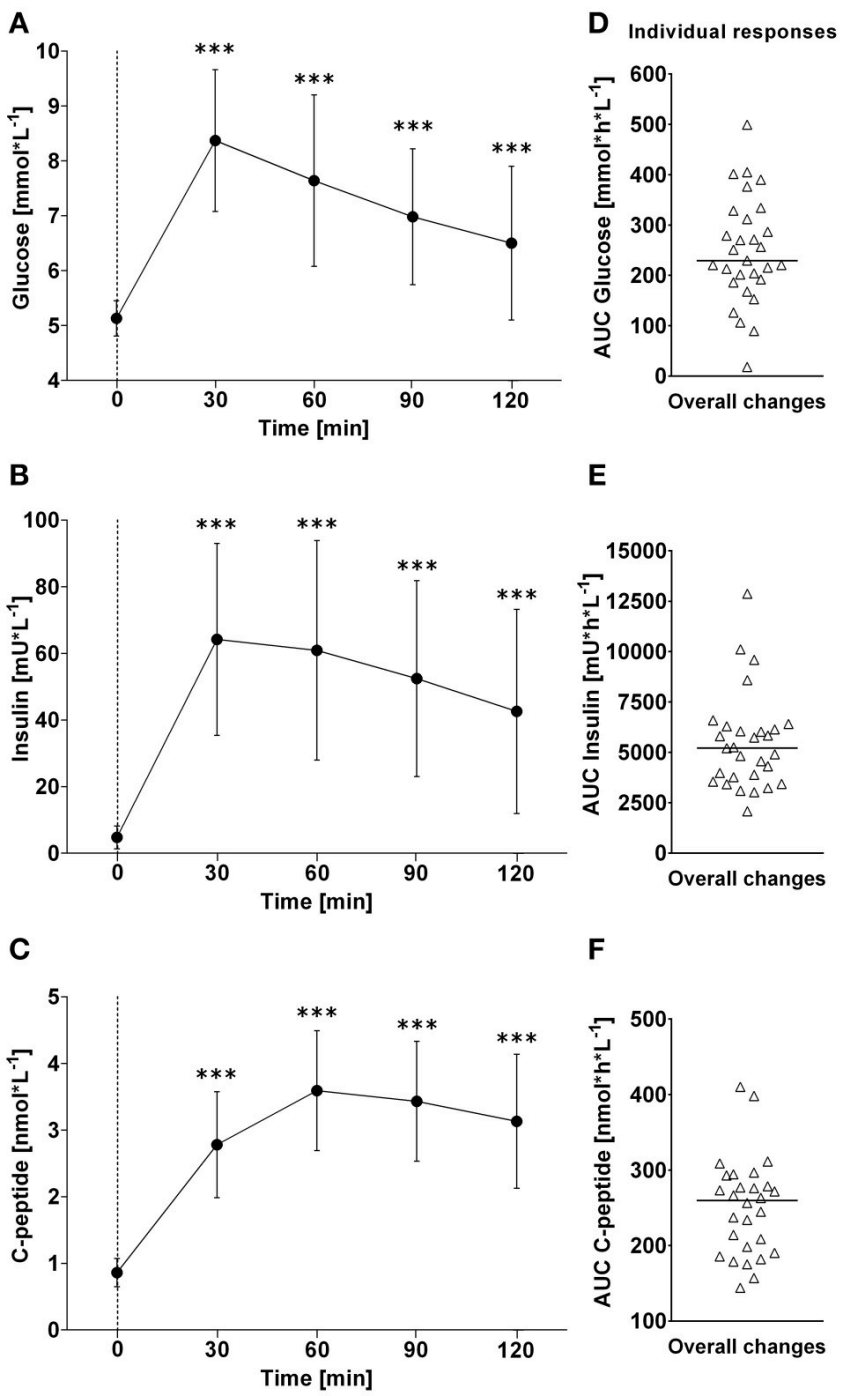
**(A–C)**: Time course of changes (Δ) from baseline in blood glucose, insulin and C-peptide levels, respectively. Closed circles (•) represent averaged data from the respective blood-draw interval, i.e., at baseline, which is denoted as 0, and every 30 min thereafter up to 120 min. **(D–F)** Represent area under curve analysis using the trapezoid method for glucose **(D)**, insulin **(E)** and C-peptide **(F)**. ^***^P < 0.005 represents statistically significant differences over time compared to baseline values (left). **(A–C)** Values are reported as means ± SD, whilst **(D–F)** data are presented as a scatter dot plot with a median (bold horizontal dashed line).

Statistical analyses were performed using statistical software: (i) Statistix version 8.0, Analytical Software, St. Paul, MN, USA and (ii) GraphPad Prism, Version 6, GraphPad Software, Inc., La Jolla, USA. All values were reported either as means ± SD (Table 1) or as medians with or without (Figures 1–4) range and the corresponding upper and lower 95% confidence intervals (CI). Testing for normal distribution was performed using the D'Agostino & Pearson omnibus normality test. Repeated measures ANOVA with Dunnett's multiple comparison *post-hoc* testing or the Friedman test with Dunns *post-hoc* testing was used to test for changes over time from baseline level (left panels Figures 1–4). Pearson correlation analysis was used to assess associations between overall and peak changes in systolic- and diastolic BP (dependent variables) and other concurrently assessed anthropometric-, blood-, and cardiovascular variables (Tables 2, 3). Pearson's *r* statistic is used to determine the effect size when two variables are continuous and when there is only one group (i.e., no separate intervention and control) (Nakagawa and Cuthill, 2007). Given that both variables (BP and fat mass) were continuous and that the study did not have separate groups, Pearson's correlation was used as an index of effect size to support the statement of a trend throughout the manuscript. All reported *p*-values are two-sided and significance was set at *p* < 0.05.

**Table 1:** Resting baseline subject characteristics.

**Variable**	**Mean ± SD**	**Min–Max**	***n***
Fasting glucose, mmol/L	5.1 ± 0.3	4.3–5.9	29
Fasting insulin, mIU/L	4.8 ± 3.4	0.5–14.3	29
Fasting C-peptide, nmol/L	0.86 ± 0.22	0.53–1.37	28
HOMA-IR, AU	1.1 ± 0.8	0.1–3.6	29
Age, years	23.5 ± 3.3	20–33	30
Weight, kg	77 ± 12	57–104	30
Height, cm	178 ± 8	166–197	30
Body Mass Index, kg/m^2^	24.2 ± 3.0	18.9–29.8	30
Waist circumference, cm	86 ± 9	74–103	30
Total fat mass, %	16.4 ± 4.8	7.8–26.1	29
Total fat mass, kg	13.1 ± 5.6	5.2–25.1	29
Total trunk fat, %	19.8 ± 7.6	5.0–35.4	30
Total fat free mass, kg	64 ± 8	49–81	29
Skeletal muscle mass, kg	37 ± 5	27–46	29
Systolic blood pressure, mmHg	114 ± 6	104–129	30
Diastolic blood pressure, mmHg	73 ± 5	65–82	30
Heart rate, beats/min	65 ± 8	51–83	30
Stroke volume, mL	82 ± 14	53–115	30
Cardiac output, L/min	5.3 ± 0.6	3.5–6.1	30
Total peripheral resistance, mmHg/min/L	16.7 ± 2.4	13.5–24.0	30
Heather index, 1/s^2^	0.23 ± 0.04	0.10–0.30	30
Double product, mmHg/beats/min	7385 ± 1066	5656–9915	30
Thoracic fluid content, kΩ^−1^	32 ± 5	25–49	30

*Values were obtained during the resting baseline measurement period prior to subjects undergoing the oral glucose tolerance test. Values are derived from fasting blood samples. SD, standard deviation; Min, minimum value; Max, maximum value; n, number of subjects; HOMA-IR, homeostatic model assessment of insulin resistance; AU, arbitrary units*.

**Table 2:** Pearson correlation analysis of the association between overall blood pressure responses to OGTT and other measured variables.

	**Δ SBP**			**Δ DBP**		
	***r***	***p***	**n**	***r***	***p***	***n***
HOMA-IR	−0.12	0.55	29	−0.21	0.28	29
Fasting glucose	+0.22	0.26	29	−0.02	0.91	29
Fasting insulin	−0.13	0.50	29	−0.20	0.29	29
Fasting C-peptide	−0.02	0.92	28	−0.12	0.55	28
ISI-Mat	−0.11	0.58	29	+0.07	0.73	29
Body Mass Index	+0.14	0.45	30	−0.14	0.45	30
WC	+0.24	0.20	30	−0.04	0.85	30
Total fat mass, %	+0.32	0.09	29	−0.09	0.64	29
Total fat mass, kg	+0.29	0.12	29	−0.09	0.65	29
Fat free mass	+0.03	0.87	29	−0.07	0.73	29
Fat mass index	+0.27	0.15	29	−0.13	0.51	29
Fat free mass index	−0.10	0.62	29	−0.20	0.30	29
SMM	+0.02	0.92	29	−0.09	0.65	29
Total trunk fat	+0.27	0.15	29	−0.01	0.95	29
Δ Systolic BP	–	–	30	+0.70	<0.005^***^	30
Δ Diastolic BP	+0.70	<0.005^***^	30	–	–	30
Δ Heart rate	+0.03	0.88	30	+0.06	0.77	30
Δ Stroke volume	−0.10	0.59	30	−0.12	0.54	30
Δ Cardiac output	−0.12	0.54	30	−0.10	0.58	30
Δ TPR	+0.22	0.25	30	+0.25	0.18	30
Δ Double product	+0.58	<0.005^***^	30	+0.43	0.02^*^	30
Δ Heather index	−0.10	0.60	30	−0.11	0.55	30

*Values are derived from a Pearson linear correlation analysis between overall systolic (Δ SBP) (i.e., averaged response over 120 min with fasting baseline values subtracted) and diastolic blood pressure (Δ DBP) responses and blood-, anthropometric-, and cardiovascular data obtained from healthy male adults who underwent an oral glucose tolerance test (OGTT). r, correlation coefficient; n, number of subjects; HOMA-IR, homeostatic model assessment of insulin resistance; ISI-Mat, Matsuda index of insulin sensitivity; WC, waist circumference; SMM, skeletal muscle mass; BP, blood pressure; TPR, total peripheral resistance; p < 0.05 was considered a statistically significant association between independent and dependent variables*.

**Table 3:** Pearson correlation analysis of the association between peak blood pressure responses to OGTT and other measured variables.

	**Δ Peak SBP**			**Δ Peak DBP**		
	***r***	***p***	**n**	***r***	***p***	***n***
HOMA-IR	+0.00	0.98	29	−0.08	0.69	29
Fasting glucose	+0.09	0.64	29	+0.04	0.85	29
Fasting insulin	+0.02	0.91	29	−0.06	0.76	29
Fasting C-peptide	+0.06	0.77	28	+0.01	0.95	28
ISI-Mat	−0.39	0.04^*^	29	−0.12	0.53	29
Body Mass Index	+0.10	0.59	30	−0.11	0.57	30
WC	+0.19	0.31	30	+0.04	0.83	30
Total fat mass, %	+0.27	0.16	29	−0.05	0.81	29
Total fat mass, kg	+0.19	0.33	29	−0.08	0.68	29
Fat free mass	−0.05	0.78	29	−0.06	0.75	29
Fat mass index	+0.18	0.34	29	−0.12	0.52	29
Fat free mass index	−0.20	0.29	29	−0.29	0.13	29
SMM	−0.05	0.76	29	−0.08	0.68	29
Total trunk fat	+0.11	0.56	29	+0.02	0.91	29
Δ Systolic BP	–	–	30	+0.62	<0.005^***^	30
Δ Diastolic BP	+0.62	<0.005^***^	30	–	–	30
Δ Heart rate	+0.11	0.56	30	+0.04	0.83	30
Δ Stroke volume	−0.26	0.17	30	−0.28	0.14	30
Δ Cardiac output	−0.06	0.75	30	−0.13	0.50	30
Δ TPR	+0.14	0.45	30	+0.19	0.31	30
Δ Double product	+0.36	0.053	30	0.21	0.26	30
Δ Heather index	−0.10	0.61	30	−0.15	0.43	30

*Values are derived from a Pearson linear correlation analysis of peak (i.e., derived from the maximum response averaged over a 10 min interval with fasting baseline values subtracted) systolic (Δ Peak SBP) and diastolic blood pressure (Δ Peak DBP) responses and blood-, anthropometric-, and cardiovascular data obtained from healthy male adults who underwent an oral glucose tolerance test (OGTT). r, correlation coefficient; n, number of subjects; HOMA-IR, homeostatic model assessment of insulin resistance; ISI-Mat, Matsuda index of insulin sensitivity; WC, waist circumference; SMM, skeletal muscle mass; BP, blood pressure; TPR, total peripheral resistance; p < 0.05 was considered as a statistically significant association between independent and dependent variables*.

## Results

### Baseline characteristics

A total of 30 healthy, Caucasian male subjects were included in this study. Baseline resting blood sample -, anthropometric-, and cardiovascular data prior to undergoing OGTT are presented in Table 1. None of the subjects presented with T2DM, hypertension, or obesity (Table 1).

### Cardiovascular variables–overall time course changes

In response to OGTT, overall time course changes for systolic BP and diastolic BP did not change significantly over the entire observation period (Figure 1, left panels). By contrast, there was a gradual, significant increase in heart rate, with peak responses observed around 80 min post-drink (5.4 ± 2.7 beats/min), which plateaued thereafter (Figure 1, left panels). Stroke volume and cardiac output increased significantly following ingestion of the glucose drink; stroke volume peaked around 40 min post-drink (5.2 ± 5.8 ml) and returned afterwards slowly toward baseline, whilst cardiac output responses continued to plateau until the end of the study period (Figure 2, left panels). Total peripheral resistance decreased significantly during OGTT and was found to be a negative mirror image of cardiac output with a gradual decrease below baseline levels soon after glucose ingestion. This negative total peripheral resistance response was maintained for the entire study period (Figure 2, left panels). Soon after glucose ingestion, Heather index and double product began to increase above baseline values and plateaued thereafter; double product responses were more gradual than those of Heather index (Figure 3, left panels). On the other hand, thoracic fluid content increased gradually, reached significance after 80 min and peaked around 110 min post-drink (0.37 ± 0.57 kΩ^−1^) (Figure 3, left panel).

### Cardiovascular variables–individual overall and peak changes

Individual overall changes ranged from +0.3 to +7.5 beats/min (Median: +3.6; 95% CI from +2.7 to +4.8 beats/min) for heart rate, from −4.9 to +4.7 mmHg (Median: +0.0; 95% CI from −0.8 to +1.5 mmHg) for systolic BP, and from −4.5 to +2.8 mmHg (Median: −0.4; 95% CI from −1.9 to +0.2 mmHg) for diastolic BP (Figure 1, right panels). Individual overall changes ranged from −8.2 to +8.8 ml (Median: +3.2; 95% CI from +2.3 to +3.9 ml) for stroke volume, from −0.44 to +0.92 L/min (Median: +0.48; 95% CI from +0.39 to +0.79 L/min) for cardiac output, and from −3.3 to +1.8 mmHg/min/L (Median: −1.7; 95% CI from −1.9 to −1.5 mmHg/min/L) for total peripheral resistance (Figure 2, right panels). Finally, individual overall changes ranged from −0.03 to +0.08 1/s^2^ (Median: +0.03; 95% CI from +0.03 to +0.04 1/s^2^) for Heather index, from −178 to +970 mmHg/beats/min (Median: +409; 95% CI from +298 to +611 mmHg/beats/min) for double product, and from −0.47 to +0.69 kΩ^−1^ (Median: 0.15; 95% CI from +0.01 to +0.30 kΩ^−1^) for thoracic fluid content (Figure 3, right panels).

Individual peak changes ranged from +3.3 to +14.1 beats/min (Median: +7.1; 95% CI from +6.3 to +9.4 beats/min) for HR, from −1.3 to +9.5 mmHg (Median: +4.8; 95% CI from +2.4 to +5.2 mmHg) for systolic BP, and from −0.9 to +7.0 mmHg (Median: +9.0; 95% CI from +5.3 to +10.2 mmHg) for diastolic BP (Figure 1, right panels). Individual peak changes ranged from −0.4 to +18.4 ml (Median: +3.2; 95% CI from +2.3 to +3.9 ml) for SV, from −0.12 to +2.08 L/min (Median: +0.91; 95% CI from +0.64 to +1.07 L/min) for cardiac output, and from −2.6 to +3.3 mmHg/min/L (Median: −0.33; 95% CI from −0.24 to −0.54 mmHg/min/L) for total peripheral resistance (Figure 2, right panels). Finally, individual peak changes ranged from +0.00 to +0.16 1/s^2^ (Median: +0.06; 95% CI from +0.05 to +0.08 1/s^2^) for Heather index, from +417 to +1,998 mmHg/beats/min (Median: +1,090; 95% CI from +754 to +1,314 mmHg/beats/min) for double product, and from +0.02 to +1.89 kΩ^−1^ (Median: +0.46; 95% CI from +0.28 to +0.76 kΩ^−1^) for thoracic fluid content (Figure 3, right panels).

### Blood sample variables–overall time course changes and individual responses

In response to OGTT, glucose and insulin increased significantly above baseline levels and peaked around 30 min post-drink (Glucose: +8.4 ± 1.3 mmol/L, +6.2 to +10.6 (min to max) mmol/L; Insulin: +64 ± 29 mIU/L, +26 min to +124 max mIU/L). C-peptide increased gradually and significantly above baseline levels, and peaked around 60 min post-drink (+3.6 ± 0.8 nmol/L +1.2 to +4.3 (min to max) nmol/L), plateauing thereafter (Figure 4, left panels).

Individual overall changes ranged from +18 to +500 mmol/L/h (Median: +230; 95% CI from +202 to +287 mmol/L/h) for glucose, from +2,075 to +12,870 mIU/L/h (Median: +5,205; 95% CI from +3,884 to 6,042 mIU/L/h) for insulin, and from +144 to +410 nmol/L/h (Median: +260; 95% CI from +226 to +276 nmol/L/h) for C-peptide (Figure 4, right panels).

### Linear correlation analysis

Overall changes in systolic BP correlated with overall changes in diastolic BP (*r*: +0.70, *p* < 0.005) and double product (*r*: +0.58, *p* < 0.005), but not with glycemic parameters (Table 2). Aside from a trend toward a significant association with total fat mass [%] (*r*: +0.32, *p* = 0.09), overall changes in systolic BP did not correlate with anthropometric parameters (Table 2). Overall changes in diastolic BP correlated positively with overall changes in double product (*r*: +0.43, *p* = 0.02), but with no other parameter (Table 2).

Peak changes in systolic BP correlated with peak changes in diastolic BP (*r*: +0.62, *p* < 0.005) and weakly with the ISI-Mat (*r*: −0.39, *p* = 0.04), but not with other glycemic parameters (Table 3). Peak changes in diastolic BP did not correlate with any investigated parameter (Table 3).

## Discussion

Tightly controlled human studies on BP changes in response to glucose drinks are scarce and the factors underlying how glucose ingestion could affect BP regulation are not fully understood. Therefore, we evaluated continuously beat-to-beat cardiovascular and glycemic blood parameter responses to a standardized 75 g oral glucose drink. Additionally, and given the important influence of body composition on the cardiovascular and metabolic system, we evaluated anthropometric and adiposity parameters in order to explore their potential association with overall and peak changes in BP. We clearly observed a divergent effect of glucose with substantial inter-subject variability for overall systolic BP measurements where 50% of subjects either increased or decreased BP in response to glucose ingestion with an effect of insulin sensitivity or body composition. With respect to peak changes in systolic BP, however, the aforementioned two-face scenario was no longer present, but rather a prevailing increase in BP where 63% increased peak systolic BP by more than a clinically relevant 4 mmHg; a potential effect of insulin sensitivity was observed, but not body composition. Therefore, our data provide evidence that glucose ingestion substantially elevates systolic BP over a short period of time, a response that is weakly associated with insulin sensitivity but not with body composition parameters.

Despite accumulating scientific evidence from cross-sectional studies (Brown et al., 2011) and randomized controlled trials (Raben et al., 2002) regarding the potential impact of chronic consumption of sugar-sweetened beverages on BP, the underlying mechanisms for the impact of sugary drinks on BP regulation remain elusive. The notion was put forward more than a decade ago that repeated acute increases in BP, evoked by a postprandial state due to the ingestion of sugary beverages, could predispose to the development of CVD (Dickinson and Brand-Miller, 2005). In randomized controlled trials, it has repeatedly been shown that fructose, but not glucose or sucrose, increased BP in healthy young adults (Brown et al., 2008a; Grasser et al., 2014). Moreover, the glucose moiety of sucrose seems to potentially counteract fructose-induced increases in BP (Grasser et al., 2014). However, the notion that glucose does not affect BP stands in contrast to findings from a cross-sectional study where direct associations of glucose intake with BP were observed (Brown et al., 2011). In addition to the limitations of interpreting findings from such cross-sectional studies (Carlson and Morrison, 2009), another potential explanation for the observed difference could be the large inter-subject variability, where subjects either increase or decrease BP in response to a glucose-containing drink. Indeed, a *post-hoc* analysis of data from a previously published randomized controlled trial found substantial inter-subject variability in the BP response to glucose ranging from −2.3 to +8.3 mmHg (averaged over 60 min post-drink after subtracting baseline values) (Grasser et al., 2014). Notwithstanding the lack of significant overall change in systolic BP and diastolic BP in response to glucose ingestion, our present study observed a large inter-subject variability in overall and peak (as a surrogate of treatment-induced changes of short-term BP variability Mancia, 2012) BP changes, which contributes to the growing scientific evidence on reproducible systolic BP changes in humans. Moreover, 63% increased peak systolic BP by more than 4 mmHg. Similar findings were observed, but to a lesser degree, for overall and peak diastolic BP changes. Our findings emphasize the importance of focusing on inter-subject variability, rather than on mean results, in order not to neglect potential treatment responders. In the context of such treatment responders, our reported BP findings here in response to a standardized glucose ingestion could have potential implications for the development of a screening tool where susceptible individuals are followed over a longer period of time in order to monitor the potential emergence of hypertension.

It is worth mentioning that impaired glucose tolerance, a condition that usually precedes T2DM, substantially elevates the risk of CVD (DeFronzo and Abdul-Ghani, 2011). Therefore, the possibility arises that even early stage perturbations in glucose metabolism may affect the cardiovascular system and that glycemic changes in response to a glucose drink could affect BP regulation. In contrast to the impact of insulin on total peripheral resistance and cardiac output in response to glucose ingestion in normal glucose tolerance, it is suggested that in a state of impaired glucose tolerance, which is characterized by elevated resting insulin and sympathetic neural activity (Rowe et al., 1981), an additional surge in insulin will further raise sympathetic nerve activity and, therefore, impact on total peripheral resistance (Ferrannini et al., 1997). In addition to the contention by Ferrannini et al. (1997), which suggested that insulin resistance could lead to a reduced vasodilator response that in turn raises BP (Ferrannini et al., 1997), we speculate that a state of impaired glucose tolerance proportionately affects cardiac parameters, i.e., heart rate and inotropy, to a greater extent than the vasodilatory effects. This could, in turn, raise BP due to the augmented effect of a raised sympathetic tone on cardiac cells rather than on peripheral vasodilation. Further studies are warranted to investigate this important contention in light of the fact that T2DM etiology has frequently been associated with the emergence of hypertension.

Using an OGTT in order to investigate a potential association between insulin and BP, Haffner et al. (2002) observed a weak relationship between fasting glycemic parameters (insulin and HOMA IR) and BP, which were similar between different ethnicities. However, apart from a weak association (*r* = +0.39) between peak changes in systolic BP and ISI-Mat, as a measure of insulin sensitivity (Matsuda and DeFronzo, 1999), we did not observe any further association between glycemic parameters, and indices of glucose metabolism (HOMA-IR), with overall and peak changes in systolic BP. Our findings are in agreement with a previous study where variations in HOMA, as a marker of insulin sensitivity, did not explain BP in men (Poirier et al., 2005). In this context, the ISI-Mat has been shown to be a better predictor for the assessment of hypertension risk compared to the HOMA-IR (Furugen et al., 2012). Moreover, Ferrannini (Ferrannini et al., 1997) investigated, in non-diabetic men and women, the impact of insulin resistance on BP parameters using an euglycemic insulin clamp technique at physiological insulin concentrations and observed in a lean subgroup an inverse association between insulin sensitivity (*M*-value) and systolic BP, which is in line with our findings. Taken together, our results suggest that in healthy, non-obese, adult men, insulin sensitivity potentially affects systolic BP, but not diastolic BP, in response to a glucose drink.

Another potential contributor to increasing BP in humans is the distribution of adipose tissue stores, where abdominal adipose tissue in particular has been suggested to be a key correlate of health risks associated with being overweight or obese (Tchernof and Després, 2013). In a recent observational study, where a hypothesized relationship between visceral adipose tissue, quantified by a 1.5 T magnetic resonance imaging scanner, and incident hypertension was investigated, increasing levels of visceral fat were associated with increasing risk for emergence of hypertension (Chandra et al., 2014). In our study, overall and peak systolic and diastolic BP changes were not significantly related to any body composition parameter investigated in this study. However, total fat mass [%] and total trunk fat [%], which has been validated against magnetic resonance imaging for the prediction of abdominal fat percentage (Browning et al., 2010), showed a trend toward a positive association with overall changes in systolic BP (*r* = +0.32, *p* = 0.09). It is possible to speculate that a larger sample size in our study may have resulted in a significant *p*-value along with an unchanged correlation coefficient (Altman and Krzywinski, 2015).

**Figure 5:**
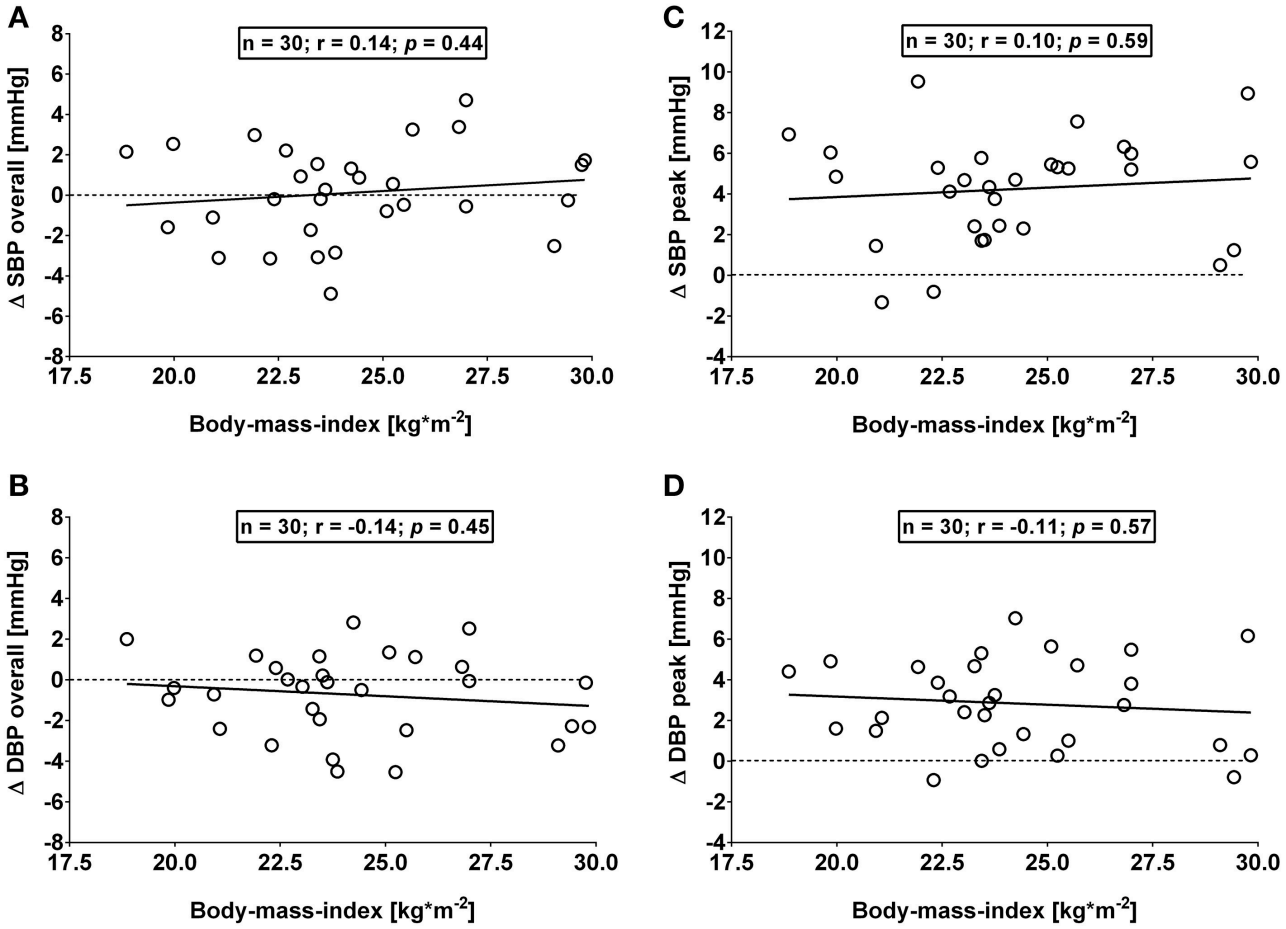
**(A,B)**: Correlation analysis of overall (i.e., averages over 120 min with baseline values subtracted) changes in systolic (Δ SBP) and diastolic blood pressure (Δ DBP) responses and body mass index. **(C,D)**: Correlation analysis of peak (i.e., derived from the maximum response averaged over a 10 min interval) changes in systolic (Δ SBP) and diastolic blood pressure (Δ DBP) responses and body mass index. Data derived from healthy male adults who underwent an oral glucose tolerance test. *n* = number of subjects; *r*: correlation coefficient; *p* < 0.05 was considered as a statistically significant association between variables.

This study has a number of caveats: firstly, (i) use of the Task Force Monitor for BP and stroke volume measurements was not validated in the current study. However, the continuous non-invasive arterial BP technology integrated in the Task Force Monitor has previously been validated against clinically invasive gold standards and was found to be comparable in terms of continuity, accuracy and waveform dynamics (Jeleazcov et al., 2010; Ilies et al., 2012). A second potential limitation of the study (ii) is the fact that recruitment was confined to a Caucasian subpopulation in order to avoid potential confounding as a result of ethnic differences in the OGTT response (Sleddering et al., 2014), which limits the generalizability of our findings. Thirdly, (iii) the clinical significance and relevance of a short-term 4 mmHg change in BP may be questioned. In this context, even short-term elevations in BP, if repeated over a long time, could have the potential to affect the cardiovascular system in a cumulative fashion (Brown et al., 2008b). Moreover, a growing body of literature exists on the vascular effects consequent to T2DM (Montero et al., 2013; Tousoulis et al., 2013), therefore, we deem any potential vascular effect of a glucose load to be noteworthy. In this context, it is important to bear in mind that prolonged sitting, which has previously been shown to be associated with endothelial dysfunction (Restaino et al., 2016), could be suggested to contribute to the variability in BP response in our study where subjects sat continuously for > 3 h. In addition, since gastric emptying accounts for up to 34% of the variation in peak plasma glucose (Horowitz et al., 1993), interindividual differences in gastric emptying in this study could be suggested to account for some of the variability in the observed BP response. A final caveat (iv) can be found in not separating our cohort based on their body mass index in a normal weight and an overweight group. However, our observed overall and peak BP responses did not differ significantly (all *p* > 0.10) between proposed groups when separated by body mass index, i.e., Group A: Body mass index from 18.5 to 24.9 kg/m^2^ (mean: 22.5 ± 1.6 kg/m^2^; *n* = 19) and Group B: Body mass index from 25.0 to 29.9 kg/m^2^ (mean: 27.3 ± 1.9 kg/m^2^; *n* = 11), therefore we combined both groups for the final analysis. Moreover, no significant association was observed for overall and peak changes in BP and body mass index (Figures 5A–D). In this context, the current study also did not include obese subjects. The lack of correlation between systolic BP and body composition, as well as the weak association with insulin sensitivity could have been different in an obese group where a stronger correlation between fat mass and insulin sensitivity would be expected based on previous studies (McLaughlin et al., 2011). However, we observed a trend toward an association between overall changes in systolic BP and total fat mass in percentage (*r* = +0.32, *p* = 0.09). Moreover, body fat mass in percentage and insulin sensitivity correlated in our study (*r* = −0.59, *p* < 0.005), which is in agreement with the findings by McLaughlin and colleagues (McLaughlin et al., 2011).

In conclusion, our data show that ingestion of 75 g of glucose elevates BP in a healthy non-obese population. We observed two faces to the effect of glucose on BP: overall changes in systolic BP were increased in half of the subjects, while in the other half, overall changes were decreased. This divergent effect was not observed for peak changes in systolic BP, which were increased in > 90% of subjects in response to glucose ingestion. Changes in BP do not appear to be dependent on body composition, but may be influenced to some extent by insulin sensitivity. In addition, a large variability in the response to glucose was observed for all variables, whether directly or indirectly measured or calculated. This large variability highlights the need for great caution when interpreting the average findings from one single study, particularly studies with small sample sizes. Further work is required to identify factors associated with this variability in the BP response to glucose ingestion and the potential influence of insulin sensitivity.

## Author contributions

CM was fully responsible for the organization of the study including screening and recruitment, anthropometric measurements, and processing and preparation of blood samples for subsequent analysis. BF and IS analyzed the processed blood sample aliquots. EG designed the study, performed venipuncture and cannulation, performed cardiovascular measurements, and wrote the first draft of the manuscript. EG performed statistical analyses. All study authors had full access to the final statistical data and contributed toward the study manuscript.

### Conflict of interest statement

The authors declare that the research was conducted in the absence of any commercial or financial relationships that could be construed as a potential conflict of interest.
